# Recognising and Managing Refractory Coeliac Disease: A Tertiary Centre Experience

**DOI:** 10.3390/nu7125506

**Published:** 2015-12-01

**Authors:** Ikram Nasr, Iman Nasr, Carl Beyers, Fuju Chang, Suzanne Donnelly, Paul J. Ciclitira

**Affiliations:** 1 Gastroenterology Department, Guys and St Thomas’ Hospital, Westminster Bridge Road, London SE1 7EH, UK; ikramnasr@hotmail.com (I.N.); suzannedonnelly@nhs.uk (S.D.); 2 Allergy and Immunology Department, Pathology and Pharmacy Building, Royal London Hospital, 80 Newark Street, London E1 2ES, UK; drimannasr@gmail.com; 3 Department of Pathology, Guys and St Thomas’ Hospital, Westminster Bridge Road, London SE1 7EH, UK; Carl.Beyers@viapath.co.uk (C.B.); fuju.chang@gstt.nhs.uk (F.C.)

**Keywords:** non-responsive coeliac disease (NRCD), refractory coeliac disease (RCD), gluten free diet (GFD), enteropathy associated T-cell lymphoma (EATL), ulcerative jejunitis, villous atrophy, T-cell receptor (TCR), clonality, polymerase chain reaction (PCR), intra-epithelial cell lymphocytes (IEL)

## Abstract

Refractory coeliac disease (RCD) is a rare complication of coeliac disease (CD) and involves malabsorption and villous atrophy despite adherence to a strict gluten-free diet (GFD) for at least 12 months in the absence of another cause. RCD is classified based on the T-cells in the intra-epithelial lymphocyte (IEL) morphology into type 1 with normal IEL and type 2 with aberrant IEL (clonal) by PCR (polymerase chain reaction) for T cell receptors (TCR) at the β/γ loci. RCD type 1 is managed with strict nutritional and pharmacological management. RCD type 2 can be complicated by ulcerative jejunitis or enteropathy associated lymphoma (EATL), the latter having a five-year mortality of 50%. Management options for RCD type 2 and response to treatment differs across centres and there have been debates over the best treatment option. Treatment options that have been used include azathioprine and steroids, methotrexate, cyclosporine, campath (an anti CD-52 monoclonal antibody), and cladribine or fluadribine with or without autologous stem cell transplantation. We present a tertiary centre’s experience in the treatment of RCD type 2 where treatment with prednisolone and azathioprine was used, and our results show good response with histological recovery in 56.6% of treated individuals.

## 1. Introduction

Enteropathy related to coeliac disease (CD) occurs in genetically predisposed individuals upon exposure to toxic gluten resulting in various gastrointestinal and extra-gastrointestinal manifestations [1]. The symptoms include diarrhoea, bloating, symptoms of malabsorption and anaemia. Diagnosis is based on a positive coeliac serology in addition to duodenal biopsies, which can demonstrate villous atrophy and increased intraepithelial lymphocytes. The latter is considered the gold standard for diagnosis [2]. Most cases respond to a strict elimination of gluten from the diet, which is currently the only accepted treatment for CD. However, a group of patients may continue to exhibit symptoms despite treatment and some describe this as non-responsive coeliac disease (NRCD). The majority of NRCD is related to ongoing gluten ingestion, but in some other cases, the symptoms are not related to coeliac disease and investigation for an alternative diagnosis is recommended. Less frequently, the ongoing symptoms of malabsorption in patients with confirmed CD is secondary to refractory coeliac disease.

RCD is divided into primary (absent response to gluten-free diet (GFD)) or secondary (previously responded to GFD but has now relapsed) [3]. Another classification is type 1 and type 2 based on the clonality of the T-cell receptors. Differentiating between RCD type 1 and RCD type 2 is not easy and requires experience and good diagnostic services. It is necessary to recognize and manage RCD type 2, which has a less predicted response and a poor prognosis due to the associated complications including ulcerative jejunitis and enteropathy associated T-cell lymphoma (EATL). Diagnosing RCD type 1, RCD type 2, ulcerative jejunitis and EATL is frequently complex, requiring small intestinal biopsy histology, intra-epithelial lymphocyte (IEL) phenotype and morphology, and T-cell receptor (TCR) clonality testing using PCR to aid the diagnosis (Table 1) [4]. Treatment options vary due to the low incidence of RCD type 2 resulting in small numbers of randomized clinical trials. Prednisolone combined with a thiopurine has been used in some centres for treatment of RCD type 1 and RCD type 2 with good success [3]. There has been a reported clinical improvement in 75% of patients with RCD type 2 [5]. Malamut *et al.* observed a histologic response in some of the few cases with RCD type 2 following treatment with methotrexate or anti-tumor necrosis factor α [6]. Treatment with cladribine (2-chlorodeoxyadenosine (2-CdA)) was studied in 32 patients and a response noted in 18 cases with a statistically significant increase in survival. Alemtuzumab (an anti CD-52 monoclonal antibody) has been used in single or limited cases with variable success [7,8].

**Table 1:** Comparison between refractory coeliac disease (RCD) type 1, RCD type 2, ulcerative jejunitis and enteropathy associated T-cell lymphoma (EATL).

Investigations	RCD Type 1	RCD Type 2	Ulcerative Jejunitis	EATL
**Histopathology**	Identical to any Marsh classification of coeliac disease	Marsh ≥ II	Mucosal ulceration with villous atrophy and IEL in adjacent mucosa.	Infiltration of medium-sized or large pleomorphic lymphoid cells
**Intraepithelial lymphocyte (IEL) phenotype**	>70% IEL are surface CD3+ and CD8+	Majority have an aberrant IEL CD3+/CD8− phenotype Rarely have normal CD3+ and CD8+	Mucosal ulceration with villous atrophy and IEL in adjacent mucosa.	Neoplastic cells are CD3+ and large cell variant are CD30+ Background IELs are mostly phenotypically abnormal (CD3+/CD8−)
**T-cell receptor gamma gene rearrangement PCR**	Polyclonal	Monoclonal	Monoclonal	Monoclonal

We report a single centre retrospective study of all cases of RCD type 2 using the coeliac database in a single centre between 2000 and 2015. We have concluded that Prednisolone combined with azathioprine can be used successfully to treat RCD type 2. Our experience shows it is a safe and successful approach to improve prognosis.

## 2. Methods

We reviewed the cases of RCD with negative coeliac serology retrospectively over a period of 15 years from 2000 to 2015. The information was collected from patient case notes and the hospital electronic patient records. Thirty-seven patients were diagnosed with RCD type 2 (59% female). The age range was 30–87 (mean age 58). We excluded 7 patients from the study: one was a recent diagnosis and was yet to commence treatment, 2 were diagnosed with RCD type 2 and referred to our centre, but we diagnosed established EATL, one had major comorbidities and opted not to start treatment, and 3 relocated abroad. The human leucocyte antigen (HLA) calls II gene, or HLA-DQ2, which is known to have a strong association with coeliac disease, was found in 86% of the cases. The patients with RCD type 2 (*n* = 30), were treated with azathioprine and prednisolone (*n* = 27). The other patients did not tolerate azathioprine and/or prednisolone or had side effects and were given alternative treatment with thioguanine (*n* = 1), methotrexate (*n* = 1) or mycophenolate mofetil (*n* = 1). The initial dose of prednisolone we used was 20 mg daily which is reduced to 15 mg/day, and if necessary to 10 mg/day, if the patients experience side effects. The standard dose of azathioprine used was 2–2.5 mg/Kg per day, but we checked the thiopurine methyltransferase (TPMT) levels to adjust the dose if necessary depending on the patient’s methylation activity. Duodenal biopsies were immunostained and PCR of the TCR was performed. The molecular signature of the clones in each repeat biopsy was compared. We looked at the patient clinical outcome after follow up as (1) improvement or (2) remains RCD type 2 on ongoing treatment. We define improvement as conversion from RCD type 2 to RCD type 1 or responsive coeliac disease as indicated by improved symptoms of coeliac disease and malasborption in addition to evidence of downgrading of RCD type 2, including: improved histological Marsh criteria to less than 2, improved CD8 positivity on immunohistochemistery or change of TCR from monoclonal to polyclonal.

## 3. Results

**Figure 1:**
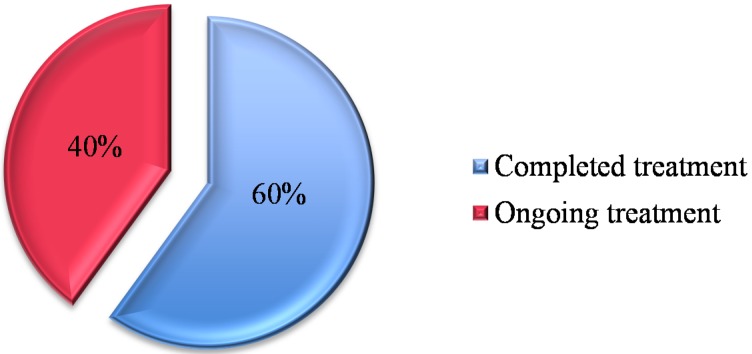
Refractory coeliac disease type 2 on treatment.

**Figure 2:**
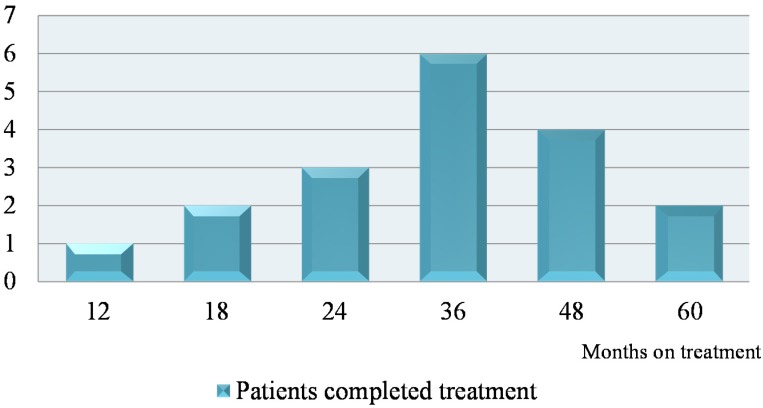
Duration of treatment in patients successfully treated.

Eighteen out of 30 patients (60%) completed treatment (Figure 1) and demonstrated improvement as summarized in Table 2. Although the polyclonality was not demonstrated in all the 18 patients, those who completed treatment with improved histological features but remained with a clonal γ-TCR population no longer demonstrated persistent clones (Table 3). The average duration of treatment was 18 to 60 months; 67% were treated for at least 36 months (Figure 2). Four patients were treated for 4 years and two patients required 5 years of treatment. The remaining 12 patients (40%) are on ongoing treatment (Table 4). The duration of treatment ranges between 12 and 78 months.

**Table 2:** Baseline and post-treatment follow up data for patients with refractory coeliac disease type 2 who completed treatment.

Gender	Age	PRE-TREATMENT							TREATMENT		POST TREATMETN							Clinical Outcome
		Histology Marsh Grade	IEL Phenotype	T-Cell Receptor Status	Hb (g/dL)	Albumin (g/L)	B12 (ng/L)	Folate (µg/L)	Treatment	Time from Treatment (Months)	Histology Marsh Grade	IEL Phenotype	T-Cell Receptor Status	Hb (g/dL)	Albumin (g/L)	B12 (ng/L)	Folate (µg/L)	
**Female**	**69**	3a	CD8 + ve	Clonal	12.9	48	319	20	Azathioprine + Prednisolone	0	1	CD8 + ve	Polyclonal	13.9	52	407	3	Asymptomatic Good quality of life (QOL)
**Female**	**83**	3b	CD8 − ve	Clonal	11.7	35	294	17.3	Azathioprine + Prednisolone	6	3a	CD8 − ve	No amplification	11.6	40	495	4	Asymptomatic QOL affected by comorbidities.
**Female**	**80**	3b	CD8 − ve	Clonal	11	45	379	3.5	Azathioprine + Prednisolone	12	3a	CD8 − ve	Clonal	11.9	43	>128	4.5	Asymptomatic QOL affected by comorbidities.
**Male**	**49**	3b	CD8 + ve	Clonal	14	47	319	14.3	Azathioprine + Prednisolone	14	3a	CD8 − ve	Polyclonal	14.5	46	>128	17.2	Asymptomatic Good QOL
**Female**	**79**	3b	CD8 + ve	Clonal	14.8	47	279	4.7	Azathioprine + Prednisolone	18	3a	CD8 + ve	Polyclonal	14.9	49	>1000	7.2	Asymptomatic QOL affected by comorbidities.
**Female**	**74**	3c	CD8 − ve	Clonal	11.4	41	1500	6.9	Azathioprine + Prednisolone	20	3b	CD8 + ve	Polyclonal	NA	NA	NA	NA	Asymptomatic Good QOL
**Male**	**50**	3a	CD8 + ve	Clonal	14.2	49	177	15.4	Azathioprine + Prednisolone	21	1	CD8 + ve	Polyclonal	13.9	50	70	17.6	Asymptomatic Good QOL
**Male**	**64**	3a	CD8 − ve	Clonal	14.7	48	333	3.6	Azathioprine + Prednisolone	26	2	CD8 – ve	Polyclonal	14.9	50	500	3.4	Asymptomatic Good quality of life
**Male**	**45**	3a	CD8 − ve	Clonal	13.8	43	177	15.4	Azathioprine + Prednisolone	28	3a	CD8 + ve	Polyclonal	12.1	46	203	13.8	Asymptomatic Good QOL
**Female**	**55**	3a	CD8 + ve	Clonal	11.9	41	247	5.1	Azathioprine + Prednisolone	36	3a	CD8 + ve	Equivocal	127	44	198	5.8	Asymptomatic Good QOL
**Female**	**66**	3b	CD8 − ve 50%	Clonal	13.7	43	200	9.5	Azathioprine + Prednisolone	36	3a	CD8 + ve 75%	Equivocal	14.1	54	231	12	Asymptomatic Good QOL
**Male**	**63**	3a	CD8 − ve	Clonal	16.1	47	140	2	Azathioprine + Prednisolone	36	3a	CD8 – ve	Clonal	15.9	47	195	3.1	Asymptomatic Good QOL
**Female**	**57**	3b	CD8 + ve	Clonal	13.5	47	559	4.2	Azathioprine + Prednisolone	36	1	CD8 + ve	Polyclonal	14	46	470	18	Asymptomatic Good QOL
**Female**	**67**	3a	CD8 + ve	Clonal	13	46	696	16.8	Mycophenolate mofetil	36	3a	CD8 + ve	Polyclonal	12.9	46	128 (active B12)	5.2	Asymptomatic Good QOL
**Male**	**71**	3a	CD8 + ve 50%	Clonal	14.9	36	155	9.5	Azathioprine + Prednisolone	36	1	CD8 + ve 100%	Clonal	12.6	47	339	7.8	Asymptomatic. QOL affected by comorbidities.
**Male**	**70**	3a	CD8 − ve	Clonal	13.7	48	64 (active B12)	5.2	Azathioprine + Prednisolone	53	1	CD8 − ve 50%	Polyclonal	15	45	68 (active B12)	20	Asymptomatic Good QOL
**Female**	**84**	3b	CD8 − ve	Clonal	12.4	38	81	12.3	Azathioprine + Budesnonide	54	3b	CD8 + ve	Polyclonal	14.1	40	210	>20	Asymptomatic. QOL affected by comorbidities.
**Female**	**74**	3b	CD8 − ve	Clonal	13.7	43	287	1.9	Azathioprine + Prednisolone	60	3b	CD8 − ve 50%	Polyclonal	11.7	42	86	2.7	Asymptomatic Good QOL

**Table 3:** Persistent identical clones observed at the start of treatment and at follow up.

Treatment Outcome	Number of Patients with Identical Clones at the Start of Treatment	Number of Cases with Identical Closes at the End of Treatment or at Latest Follow up
RCD type 2 patient responded to treatment (*n* = 18)	7 patients with identical clones	0 persistent clones
RCD type 2 who remain on treatment (*n* = 12)	9 patient identical clones	9 persistent identical clones

**Table 4:** Baseline and post-treatment follow up data for patients with refractory coeliac disease type 2 who are on ongoing treatment.

Gender	Age	PRE-TREATMENT							TREATMENT		POST TREATMETN						
		Histology Marsh Grade	IEL Phenotype	T-Cell Receptor Status	Hb (g/dL)	Albumin (g/L)	B12 (ng/L)	Folate (µg/L)	Treatment	Time from Treatment (Months)	Histology Marsh Grade	IEL Phenotype	T-Cell Receptor Status	Hb (g/dL)	Albumin (g/L)	B12 (ng/L)	Folate (µg/L)
**Male**	**61**	5.2	CD8 − ve	Clonal	12.9	28	42 (active B12)	5.2	Azathioprine + Prednisolone	12	NA	NA	Equivocal	NA	NA	NA	NA
**Male**	**71**	7.5	CD8 + ve 50%	Clonal	13.2	28	18	7.5	Azathioprine + Prednisolone	13	3a	CD8 − ve	Polyclonal	15.1	41	40	>20
**Female**	**68**	3c	CD8 + ve	Clonal	14.7	47	120	13.4	Thioguanine	21	3b	CD8 + ve	Polyclonal	14.9	49	78	7.7
**Female**	**79**	3a	CD8 + ve	Clonal	14.8	47	279	4.7	Azathioprine + Prednisolone	42	1	CD8 + ve	Clonal	NA	NA	NA	NA
**Male**	**48**	3b	CD8 − ve	Clonal	11.7	33	207	3.5	Methotrexate	57	3a	CD8 − ve	Clonal	14.8	38	54	12.6
**Male**	**68**	3c	CD8 − ve	Clonal	9.7	47	1500	3.6	Azathioprine + Prednisolone	60	0	CD8 + ve	Clonal	11.3	47	1500	8.5
**Female**	**80**	3a	CD8 + ve	Clonal	12.4	38	81	12.3	Azathioprine + Prednisolone	72	0	CD8 + ve	Clonal	14.1	40	210	>20
**Female**	**64**	3a	CD8 + ve	Clonal	12.4	46	210	2.6	Azathioprine + Prednisolone	72	1	CD8 − ve 50%	Clonal	13	49	127	13.6
**Male**	**54**	3a	CD8 + ve	Clonal	14.1	46	171	4.1	Azathioprine + Prednisolone	72	3a	CD8 + ve	Polyclonal	14.8	46	70 (active B12)	7.3
**Male**	**77**	3a	CD8 + ve	Clonal	14.6	41	286	3.1	Azathioprine + Prednisolone	74	3a	CD8 + ve	Clonal	14.3	45	123	8.6
**Female**	**67**	3a	CD8 + ve	Clonal	13.7	46	157	>20	Azathioprine + Prednisolone	78	3a	CD8 − ve	Clonal	142	45	na	4.5
**Female**	**85**	3b	CD8 − ve	Clonal	13.2	46	208	8.9	Azathioprine + Prednisolone	78	2	CD8 − ve	Clonal	134	41	37	18.1

Our data show that treatment of RCD type 2 with steroids and azathioprine show good response with a histological recovery in 16 out of 30 patients (53%). The TCR clonality improved converting to polyclonal in 17/30 (56.6%). None of the patients in our cohort developed ulcerative jejunitis or EATL, providing them with a better 5-year survival.

All patients continued to be on regular follow up after completing their treatment. We observed that all patients who completed treatment were asymptomatic and the percentage of CD8 positivity improved. We also observed persistent clones in the repeat duodenal biopsies of most of the refractory cases not responsive to treatment. Of the 12 patients who have not recovered, nine had persistent identical clones on repeat biopsies. Compared to the group who have completed treatment and improved to either RCD type 1 or responsive coeliac disease (18 patients), seven had identical clones at some point during surveillance (*p*-value 0.017).

## 4. Discussion

### 4.1. Non-Responsive Coeliac Disease (NRCD)

NRCD is defined as failure of expected symptomatic response to a GFD and is diagnosed clinically and histologically. Patients frequently complain of continued symptoms including lethargy, abdominal pain and diarrhoea. Laboratory tests often exhibit low iron, B12 and folate levels [2]. On small bowel biopsy, there is evidence of incomplete small intestinal mucosal recovery, although 40% of individuals with CD on GFD will have villous atrophy for over one year [3]. In RCD, there is loss of CD8 and expression of intra-cytoplasmic CD3 by intra-epithelial lymphocytes (IEL). The prevalence of RCD ranges from 1% to 2% of patients with CD and 0.002% in the general population [9], which explains the reason for the small number of affected individuals involved in clinical trials in tertiary referral centres.

**Figure 3:**
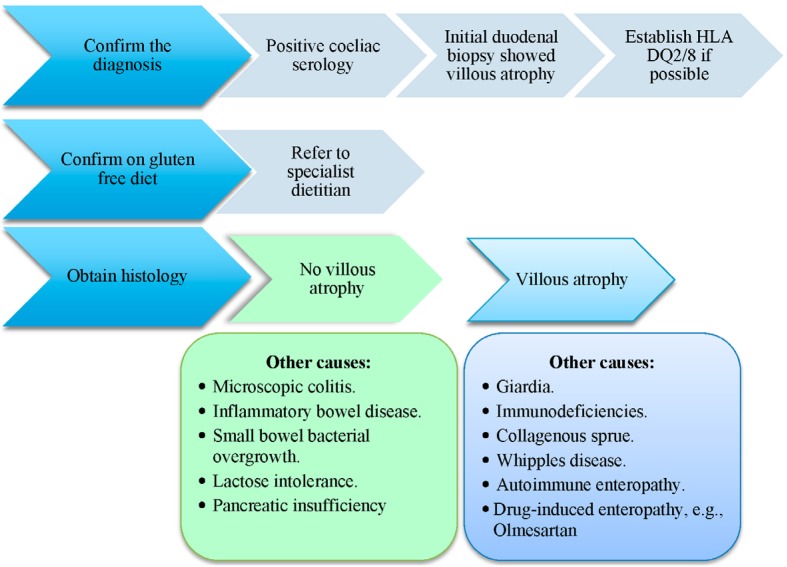
Approach to investigating non-responsive coeliac disease.

Establishing the cause for continued symptoms is the cornerstone for the management of NRCD (Figure 3). When approaching patients who remain symptomatic, it is necessary to classify them based on the history, presentation and investigations into (1) patients not adhering to a gluten-free diet; (2) a second diagnosis other than CD; (3) CD patients with complications; and (4) patients with RCD. RCD type 1 is considered if patients fail to improve after CD has been treated with GFD for one year. The most common cause of a NRCD is non-compliance with the diet or inadvertent gluten ingestion. This is estimated to occur in 90% of cases [10]. It is important when managing NRCD to bear in mind the conditions mimicking coeliac disease such as pancreatic insufficiency, lactose intolerance, small bowel bacterial overgrowth, inflammatory bowel disease, hypo-gammaglobulinemia, tropical sprue, collagenous colitis and adult onset autoimmune enteropathy. Excluding these conditions is, therefore, essential as part of the workup. This involves ensuring the individual is on a strict GFD, endoscopy with small intestinal biopsy for light microscopy, coeliac serology including IgA antibodies to tissue transglutaminase (tTG) and endomysium and polymerase chain reaction for T cell receptor monoclonality. Many centres check the immunoglobulin levels including IgA and IgG titres and subsequently test for IgG antibodies to tTG if there is IgA deficiency. Further testing includes HLA DQ2 and DQ8 status, colonoscopy, lactose and fructose intolerance, small bowel bacterial overgrowth and testing for pancreatic insufficiency. If the diagnosis of coeliac disease is confirmed, other causes for symptoms are ruled out and the patient is on a strict gluten exclusion diet, then the diagnosis of refractory coeliac disease may be entertained. Raised CD serology antibody titres imply continued gluten ingestion, either deliberate or inadvertent.

### 4.2. Refractory Coeliac Disease (RCD)

RCD is a subset of non-responsive patients with persistent or recurrent malabsorptive symptoms and signs with villous atrophy despite a strict GFD for more than 12 months [11]. Investigations will usually identify no other cause [5]. RCD is classified as primary or secondary based on the time of onset. Primary RCD is described when the individual with coeliac disease is never responsive to a GFD. On the other hand, secondary RCD occurs in patients who developed refractory coeliac having been responsive to a GFD before, months or even decades later.

The other classification is type 1 or type 2 RCD based on the phenotype of intra-epithelial lymphocytes. Type 1 RCD (RCD type 1) has a normal intra-epithelial lymphocyte phenotype whereas type 2 RCD (RCD type 2) has an abnormal clonal population. This classification is important not only to guide treatment but is of prognostic value. RCD type 2 carries a poor prognosis with a five-year mortality in RCD type 2 of about 55% [5]. RCD type 1 carries a much better prognosis with a mortality rate of 7% with aggressive treatment involving strict adherence to a GFD, nutritional support and pharmacologic intervention.

Complications of RCD include ulcerative jejunitis and enteropathy associated T-cell lymphoma (EATL), the latter being the major cause of mortality in these patients. RCD type 2 has a female preponderance with a female to male ration of 3:1, similar to responsive CD, but the ratio is reversed in EATL [6,12,13]. HLA DQ2 homozygosity is a risk factor for RCD type 2 and EATL [14].

### 4.3. Diagnosis

Diagnosis of RCD may be challenging and is in many cases a diagnosis of exclusion. Clinical assessment, pathological, histological, laboratory and radiological findings all aid in the diagnosis and enable to differentiate between the two subtypes.

In our centre, we follow a strategy when making a diagnosis of RCD type 2:

1. When making the diagnosis, the patient needs to be on a strict GFD with a negative anti-enterocyte antibody result. Dietary assessment of compliance to GFD is key and instruction and education by a specialized dietitian is advised.
2. Upper gastrointestinal endoscopy to obtain small bowel biopsies for tissue analysis. The samples are used for Marsh scoring.
3. Assessment of IEL phenotyping and polymerase chain reaction (PCR) for TCR (T-cell receptor) monoclonality at the β and/or γ loci.

The presence of abnormal (clonal) IEL in the small bowel supports a diagnosis of RCD type 2. Transient TCR clonality can be detected in patients at diagnosis and with poor compliance [15,16]. The TCR (T cell receptor) is a molecule found on the surface of T lymphocytes and is the site for antigen recognition that binds to the major histocompatibility complex (MHC)/CD-toxic gluten complex. The TCR is composed of two different protein chains which in 95% of all T cells comprises an alpha (α) and a beta (β) chain. α/β TCR are present on MHC restricted CD4+ (helper) and CD8+ (cytotoxic) T cells. In the remaining 5% these protein chains comprise the gamma and delta (γ/δ) chains, γ/δ TCR. The majority of these do not express CD4 or CD8 and are not MHC restricted. They are abundant in epithelial tissues (IEL) where they represent 10% of human intestinal intra-epithelial cells. The role of γ/δ T cells is largely unknown, although they have a number of biologic activities similar to α/β/TCR such as cytokine secretion and lysis of target cells. It is hypothesized γ/δTCR recognise antigens that are frequently encountered at epithelial boundaries between the host and external environment which may initiate immune responses before the recruitment of T cells. Not only do the histological and molecular features help differentiate between RCD type 1 and RCD type 2, but could additionally aid the diagnosis of ulcerative jejunitis and EATL (Table 1).

4. All cases of RCD type 2 should have a capsule endoscopy to exclude EATL (enteropathy associated T cell lymphoma). It is our practice if there is any suggestion of possible EATL to undertake a small bowel magnetic resonance imaging (MRI) in the first instance to exclude an obstructing lesion, which would be a contra-indication to capsule endoscopy. The capsule endoscopy should be repeated after a year to exclude the development of EATL in view of the high risk. It has been proposed that RCD type 2 should be renamed pre-EATL [17]. Double balloon enteroscopy may be required depending on the findings on capsule endoscopy in order to make a better assessment of an abnormality and obtain samples if required.
5. Cross sectional imaging including small bowel MRI, computed tomography (CT) scan and positron emission tomography (PET) scan are recommended when suspecting EATL. This can identify abnormal areas within the bowel, abnormal lymph nodes and other organ involvement.

Patients with RCD presenting with abdominal pain, weight loss or evidence of malnutrition should undergo urgent investigation. Cross-sectional imaging by CT +/− PET or MRI for the presence of lymphadenopathy or intestinal tumours should be carried out and capsule or balloon enteroscopy should be performed to diagnose any cases of EATL [18].

### 4.4. Management of RCD Type 2

Several approaches to management of RCD type 2 have been trialed in different centres (Table 5). Treatment with budesonide alone has been reported to provide a good clinical response in refractory coeliac disease, but the effects on prognosis is not clear [19,20,21,22]. Treatment with a thiopurine, including azathioprine, mercaptopurine or thioguanine, combined with prednisolone has been used in our practice with good results. Some centres report an unfavourable response to this regimen, such as the Mulder group who reported a 52% progression to EATL [5]. It is our view that factors that may affect our positive results with treatment include early detection of RCD type 2 cases, close monitoring of the patients, adherence to treatment and a multidisciplinary approach in patient care. The latter involves an expert dietitian, histopathologist with knowledge of RCD and the availability of a molecular pathology TCR PCR service, that allows close monitoring. The percentage of aberrant IEL and the percentage of clonal TCR population may additionally play a role.

There have been other reports of different treatment options, some with limited cases and variable results. Methotrexate has been used as a single agent [5] or in combination with cyclosporine [23] in a few cases with good results. Campath (anti CD-52 monoclonal antibody) has been used on single or limited cases with variable success [8]. Cladribine or fluadribine with or without autologous stem cell transplantation has been another area of interest [24]. Mulder *et al.* reported 32 patients treated with cladribine, of whom 18 had a good response [25]. Cyclosporine has also been used in RCD type 2 [26,27,28]. Wahab *et al.* reported 61% histological improvement with this treatment in a group of 13 patients with RCD type 2 [29]. There have been single cases of successful treatment with infliximab, although some of these reported cases were used to treat RCD type 1 [30,31,32]. A multicenter study on the effect of anti-TNF treatment on RCD is required to establish the value of this treatment in RCD type 2.

**Table 5:** Treatment options in RCD type 2.

Treatment Option	Recommended Dose	Outcome	References
Budesonide	9 mg (range 6–12 mg)	Good Clinical response. Also used in maintenance of clinical remission in collagenous colitis	Brar *et al.* [19] Daum *et al.* [20] Miehlke *et al.* [21] Bonderup *et al.* [22]
Combination thiopurine, including azathioprine, mercaptopurine or thioguanine, combined with prednisolone		52% progression to EATL within 4–6 years	Al-Toma *et al.* [12].
Alemtuzumab (anti CD-52 monoclonal antibody)	30 mg twice a week per 12 weeks	Not effective	Verbeek *et al*. [8].
		Effective	Vivas *et al.* [7].
Cladribine	0.1 mg/kg/day for 5 days	Thirty-two patients treated with cladribine, of whom 18 had a good response	Tack *et al.* [25].
		Six of 17 patients had clinical and histologic improvement Clinical improvement (36%), histological improvement (59%), and significant decrease in the number of clonal intraepithelial lymphocytes (35%). However, up to 41% developed EATL and died despite cladribine therapy.	Al-Toma *et al.* [33]
Cyclosporin A	5 mg/kg/day	Case report of histological and clinical improvement in a 45 year old lady with RCD type 2	Longstreth *et al.* [26].
		Single cases reported to show improvement of clinical parameters and mucosal abnormalities during treatment with cyclosporine	Bernstein *et al*. [27]. Eijsbouts *et al.* [28].
		61% histological improvement with this treatment in a group of 13 patients with RCD type 2	Wahab *et al*. [29].
Combination of pentostatin (4 mg/m^2^ every two weeks per 24 weeks) and budesonide	Pentostatin (4 mg/m^2^ every two weeks per 24 weeks)	Clinical and histological response as well as a decrease but not disappearance of clonal intraepithelial lymphocytes in 1 case	Dray *et al.* [34]
High-dose chemotherapy followed by ASCT has been explored for RCD type 2 in a pilot study from a single center		All 7 patients: Significant reduction in the aberrant T cells in duodenal biopsies associated with improvement 1 out of the 7 died of progressive neurosypillis	Al-Toma *et al.* [35]
		Out of the 4 patients with EATL, 1 patient sustained remission 32 months after ASCT. Three patients died from relapse within few months after ASCT.	Al-Toma *et al.* [36]

## 5. Conclusions

Prednisolone combined with azathioprine can be used successfully to treat RCD type 2. Our experience shows it is a safe and successful approach to improve prognosis. We successfully treated 18 out of 30 patients with RCD type 2 with this regimen, converting either to RCD type 1 or responsive coeliac disease. Where azathioprine and/or steroids are not tolerated or patients experience side effects, alternatives may be used. In our cohort, we used methotrexate (*n* = 1), thioguanine (*n* = 1) or mycophenolate mofetil (*n* = 1); the latter responded to treatment and converted to RCD type 1. Our data show improved outcomes with this management, which could be that we may be seeing affected subjects earlier through the UK tertiary referral system such that they may be less ill. In some centres, RCD type 2 patients are seen to have a serum albumin that we do not see. This suggests the patients that are referred to us are earlier in the disease process, and this may explain the improved outlook with less risk of developing EATL. In the azathioprine/steroid group, we block the aberrant immune response to gluten proteins that occurs in coeliac disease resulting in a better outcome than using other drugs with a different mechanism of action. The percentage of CD8 negative cells and/or the presence of persistent identical clones may have a role in persistent features of RCD type 2. Further studies are required to confirm this. The immunophenotyping is less sensitive than PCR TCR clonality testing. However, immunostains for CD3/CD8 are easier to be carried out in a routine pathology laboratory and almost always available in non-tertiary centres. This method can be used as a screening tool. Although the majority of IELs in RCD type 2 show an abnormal CD3+/CD8− immunophenotype, a normal CD3+/CD8+ phenotype has been seen in up to 52% of RCD type 2 cases [37]. However, some authors have questioned the reliability of this method. For instance, Goerres *et al.* found that only one out of eight RCD type 2 patients showed a loss of CD8 expression and concluded that other more sensitive ancillary tests such as flow cytometry may be necessary in the diagnosis of some RCD2 patients [19]. In flow cytometry, it is suggested that the percentage of aberrant IEL can differentiate between RCD type 1 and type 2, with type 1 exhibiting < 20% aberrant IEL and type 2 > 20%. Therefore, in our practice we used TCR clonality testing as an additional tool to confirm the diagnosis of RCD type 2. No reports appear to suggest a better prognosis of RCD2 patients with a CD3+/CD8+ immunophenotype. This could be an interesting area to explore in the future.

## References

[1] Nasr I., Nasr I.H., Ciclitira P.J. (2015). Patient management: Coeliac disease. Found. Years J..

[2] Nasr I., Leffler D.A., Ciclitira P.J. (2012). Management of celiac disease. Gastrointest. Endosc. Clin. N. Am..

[3] Woodward J. (2013). The management of refractory coeliac disease. Ther. Adv. Chronic Dis..

[4] Cellier C., Delabesse E., Helmer C., Patey N., Matuchansky C., Jabri B., Macintyre E., Cerf-Bensussan N., Brousse N., the French Coeliac Disease Study Group (2000). Refractory sprue, coeliac disease, and enteropathy-associated T-cell lymphoma. Lancet.

[5] Rubio-Tapia A., Murray J.A. (2010). Classification and management of refractory celiac disease. Gut.

[6] Malamut G., Afchain P., Verkarre V., Lecomte T., Amiot A., Damotte D. (2009). Presentation and long term follow up of refractory coeliac disease: Comparison of type I with type II. Gastroenterology.

[7] Vivas S., de Morales J., Ramos R., Suárez-Vilela D. (2006). Alemtuzumab for refractory celiac disease in a patient at risk for enteropathy-associated T-cell lymphoma. N. Engl. J. Med..

[8] Verbeek W.H.M., Mulder C.J.J., Zweegman S. (2006). Alemtuzumab for refractory celiac disease. N. Engl. J. Med..

[9] Di Sabatino A., Biagi F., Gobbi P.G., Corazza G.R. (2012). How I treat enteropathy-associated T-cell lymphoma. Blood.

[10] Dewar D.H., Donnelly S.C., McLaughlin S.D., Johnson M.W., Ellis H.J., Ciclitira P.J. (2012). Coeliac disease: Management of persistent symptoms in patients on a gluten-free diet. World J. Gastroenterol..

[11] Ludvigsson J.F., Leffler D.A., Bai J., Biagi F., Fasano A., Green P.H., Hadjivassiliou M., Kaukinen K., Kelly C.P., Leonard J.N. (2013). The Oslo definitions for coeliac disease and related terms. Gut.

[12] Al-Toma A., Verbeek M., Hadithi M., von Blomberg B., Mulder C. (2007). Survival in refractory coeliac disease and enteropathy-associated T-cell lymphoma: Retrospective evaluation of single centre experience. Gut.

[13] Rubio-Tapia A., Kelly D., Lahr B., Dogan A., Wu T., Murray J. (2009). Clinical staging and survival in refractory coeliac disease: A single center experience. Gastroenterology.

[14] Al-Toma A., Goerres M.S., Meijer J.W., Peña A.S., Crusius J.B., Mulder C.J. (2006). Human leukocyte antigen-DQ2 homozygosity and the development of refractory celiac disease and enteropathy-associated T-cell lymphoma. Clin. Gastroenterol. Hepatol..

[15] Prisco A., Troncone R., Mazzarella G., Gianfrani C., Auricchio S., Even J. (1997). Identical T-cell receptor beta chain rearrangements are present in T cells infiltrating the jejunal mucosa of untreated coeliac patients. Hum. Immunol..

[16] Ubiali A., Villanacci V., Facchetti F., Lanzini A., Lanzarotto F., Rindi G. (2007). Is TCRgamma clonality assay useful to detect early coeliac disease?. J. Clin. Gastroenterol..

[17] Nijeboer P., Malamut G., Bouma G., Cerf-Bensussan N., Koning F., van Bergen J., Cellier C., Mulder C.J.J. (2015). Therapy in RCDII: Rationale for combination strategies?. Dig. Dis..

[18] Van Weyenberg S., Meijerink M., Jacobs M., van Kuijk C., Mulder C., van Waesberghe J. (2011). MR enteroclysis in refractory coeliac disease: Proposal and validation of a severity scoring system. Radiology.

[19] Brar P., Lee S., Lewis S., Egbuna I., Bhagat G., Green P. (2007). Budesonide in the treatment of refractory celiac disease. Am. J. Gastroenterol..

[20] Daum S., Ipczynski R., Heine B., Schulzke J.D., Zeitz M., Ullrich R. (2006). Therapy with budesonide in patients with refractory sprue. Digestion.

[21] Miehlke S., Maddish A., Karimi D., Wonschik S., Kuhlisch E., Beckmann R., Morgner A., Mueller R., Greenwald R., Seitz G. (2009). Budesonide is effective in treating lymphocytic colitis: A randomized double-blind placebo-controlled study. Gastroenterology.

[22] Bonderup O.K., Hansen J.B., Birket-Smith L., Vestergaard V., Teglbjærg P.S., Fallingborg J. (2003). Long-term budesonide treatment of collagenous colitis: A randomised, double-blind, placebo-controlled trial. Gut.

[23] Malamut G., Meresse B., Verkarre V., Kaltenbach S., Montcuquet N., van Huyen J.P.D., Callens C., Lenglet J., Rahmi G., Samaha E. (2012). Large granular lymphocytic leukemia: A treatable form of refractory coeliac disease. Gastroenterology.

[24] Tack G.J., Wondergem M.J., al-Toma A., Verbeek W.H.M., Schmittel A., Machado M.V., Perri F., Ossenkoppele G.J., Huijgens P.C., Schreurs M.W.J. (2011). Auto-SCT in refractory celiac disease type II patients unresponsive to cladribine therapy. Bone Marrow Transpl..

[25] Tack G.J., Verbeek W.H., al-Toma A., Kuik D.J., Schreurs M.W., Visser O., Mulder C. (2011). Evaluation of Cladribine treatment in refractory coeliac disease type II. World J. Gastroenterol..

[26] Bernstein E.F., Whitington P.F. (1988). Successful treatment of atypical sprue in an infant with cyclosporin. Gastroenterology.

[27] Eijsbouts A.M.M., Witteman B.J.M., de Sevaux R.G.L. (1995). Undefined malabsorption syndrome with villous atrophy successfully reversed by treatment with cyclosporin. Eur. J. Gastroenterol. Hepatol..

[28] Wahab P.J., Crusius J.B., Meijer J.W., Uil J.J., Mulder C. (2000). Cyclosporin in the treatment of adults with refractory coeliac disease—An open pilot study. Aliment. Pharmacol. Ther..

[29] Dray X., Joly F., Lavergne-Slove A., Treton X., Bouhnik Y., Messing B. (2006). A severe but reversible refractory sprue. Gut.

[30] Turner S.M., Moorghen M., Probert C.S. (2005). Refractory coeliac disease: Remission with infliximab and immunomodulators. Eur. J. Gastroenterol. Hepatol..

[31] Rawal N., Twaddell W., Fasano A., Blanchard S., Safta A. (2015). Remission of refractory coeliac disease with infliximab in a pediatric patient. ACG Case Rep. J..

[32] Al-Toma A., Goerres M.S., Meijer J.W., von Blomberg B.M., Wahab P.J., Kerckhaert J.A., Mulder C.J. (2006). Cladribine therapy in refractory celiac disease with aberrant T cells. Clin. Gastroenterol. Hepatol..

[33] Longstreth G.F. (1993). Successful treatment of refractory sprue with cyclosporine. Ann. Intern. Med..

[34] Al-Toma A., Visser O.J., van Roessel H.M., von Blomberg B.M., Verbeek W.H., Scholten P.E., Ossenkoppele G.J., Huijgens P.C., Mulder C.J. (2007). Autologous hematopoietic stem cell transplantation in refractory coeliac disease with aberrant T cells. Blood.

[35] Al-Toma A., Verbeek W.H., Visser O.J., Kuijpers K.C., Oudejans J.J., Kluin-Nelemans H.C., Mulder C.J., Huijgens P.C. (2007). Disappointing outcome of autologous stem cell transplantation for enteropathy-associated T-cell lymphoma. Dig Liver Dis..

[36] Gillett H.R., Arnott I.D., McIntyre M., Campbell S., Dahele A., Priest M., Jackson R., Ghosh S. (2002). Successful infliximab treatment for steroid-refractory coeliac disease: A case report. Gastroenterology.

[37] Patey-Mariaud de Serre N., Cellier C., Jabri B. (2000). Distinction between coeliac disease and refractory sprue: A simple immunohistochemical method. Histopathology.

